# Variations in the cerebrospinal fluid dynamics of the American alligator (*Alligator mississippiensis*)

**DOI:** 10.1186/s12987-021-00248-1

**Published:** 2021-03-12

**Authors:** Bruce A. Young, James Adams, Jonathan M. Beary, Kent-Andre Mardal, Robert Schneider, Tatyana Kondrashova

**Affiliations:** 1 Department of Anatomy, Kirksville College of Osteopathic Medicine, A.T. Still University, Kirksville, MO 63501 USA; 2 Behavioral Neuroscience, Kirksville College of Osteopathic Medicine, A.T. Still University, Kirksville, MO 63501 USA; 3 Department of Mathematics, University of Oslo, Oslo, Norway USA; 4 Family Medicine, Kirksville College of Osteopathic Medicine, A.T. Still University, Kirksville, MO 63501 USA

**Keywords:** Pulsatility, Orthostatic, Myodural, Compliance, Pressure

## Abstract

**Background:**

Studies of mammalian CSF dynamics have been focused on three things: paravascular flow, pressure and pulsatility, and “bulk” flow; and three (respective) potential motive forces have been identified: vasomotor, cardiac, and ventilatory. There are unresolved questions in each area, and few links between the different areas. The American alligator (*Alligator mississippiensis*) has pronounced plasticity in its ventilatory and cardiovascular systems. This study was designed to test the hypothesis that the greater cardiovascular and ventilatory plasticity of *A. mississippiensis* would result in more variation within the CSF dynamics of this species.

**Methods:**

Pressure transducers were surgically implanted into the cranial subarachnoid space of 12 sub-adult alligators; CSF pressure and pulsatility were monitored along with EKG and the exhalatory gases. In four of the alligators a second pressure transducer was implanted into the spinal subarachnoid space. In five of the alligators the CSF was labeled with artificial microspheres and Doppler ultrasonography used to quantify aspects of the spinal CSF flow.

**Results:**

Both temporal and frequency analyses of the CSF pulsations showed highly variable contributions of both the cardiac and ventilatory cycles. Unlike the mammalian condition, the CSF pressure pulsations in the alligator are often of long (~ 3 s) duration, and similar duration CSF unidirectional flow pulses were recorded along the spinal cord. Reduction of the duration of the CSF pulsations, as during tachycardia, can lead to a “summation” of the pulsations. There appears to be a minimum duration (~ 1 s) of isolated CSF pulsations. Simultaneous recordings of cranial and spinal CSF pressures reveal a 200 ms delay in the propagation of the pressure pulse from the cranium to the vertebral canal.

**Conclusions:**

Most of the CSF flow dynamics recorded from the alligators, are similar to what has been reported from studies of the human CSF. It is hypothesized that the link between ventilatory mechanics and CSF pulsations in the alligator is mediated by displacement of the spinal dura. The results of the study suggest that understanding the CSF dynamics of *Alligator* may provide unique insights into the evolutionary origins and functional regulation of the human CSF dynamics.

## Background

The cerebrospinal fluid (CSF) system is found in all vertebrates [1, 2]. Studies in different vertebrates have identified a number of influences, or forces, acting on the CSF including: cardiac, respiratory, vasomotion, orthostatic, ciliary, transmantle gradients, and skeletal muscle. The relative importance of these different influences remains unclear, even in humans; furthermore, the functional complexity of the CSF system is still uncertain. The CSF plays a crucial role in protecting the brain from traumatic injury [3], but also maintains and regulates the brain´s chemical environment [4, 5]. Recent breakthroughs [6, 7] in neuroscience have amplified the potential regulatory capacity of the CSF system and demonstrated that neuro-degenerative diseases, and Alzheimer’s in particular, may be due to insufficient clearance of metabolic waste by the CSF. As such, it is interesting and timely to re-investigate the CSF system and its variance across species.

The forces influencing the CSF system are best known in humans. Here, as embodied by the classic Monro-Kellie doctrine [8], the out-of-phase pulsations of the arterial and venous blood flow are compensated for by CSF pulsations [9]. Furthermore, both the respiratory and vasomotor influences on the CSF have been measured by MRI [10, 11] and confirmed by modeling [12, 13]. In humans the CSF pressure varies with orthostatic (postural) pressure, likely due to a functional coupling with orthostatic changes in venous pressure [14, 15]. Finally, transmantle gradients in humans are very small, or zero [12, 16–18]. From a clinical perspective, it is known that alterations in CSF pressure can result in both physical displacement of the neural tissue as in cerebellar herniation [19], and functionally-disruptive compression of the neural tissue as in hydrocephalus [20] or spinal syrinx [21].

In humans the CSF in the intraventricular and subarachnoid spaces is freely exchanged through the median (Foramen of Magendie) and lateral (Foramen of Lushcka) apertures in the 4th ventricle. These apertures are not present in amphibians and reptiles [22]; in these species CSF continuity depends on exchange across the tela choroidea. The purpose of this study was to explore the CSF dynamics in the American alligator, *Alligator mississippiensis*, a species in which the intraventricular and subarachnoid spaces are spatially isolated. We hypothesized that the relatively restricted mobility of the CSF in *Alligator*, compared to the condition in humans, would alter the cerebral compliance and aspects of the CSF waveform dynamics. In alligators the ventilatory pattern is far more plastic than in humans or other mammals; alligators are capable of sustained apnea [23, 24], and switch between multiple ventilatory mechanics [25, 26]. Cerebral blood flow is more variable in alligators than in humans, due in part to the alligator's ability to actively redirect (shunt) cardiac output between the pulmonary and systemic circuits [27, 28]. Alligators, like some other reptiles, appear to lack a barostatic reflex [29, 30].

Thus, when compared to humans, alligators have a different cerebral compliance, and at least three of the known CSF influences (cerebral arterial pressure, ventilation, and orthostatic forces) are more variable in alligators. For these reasons, we hypothesized that the CSF dynamics in *Alligator* would be more variable than in humans, particularly with respect to the cardiac and ventilatory cycles. To explore these hypotheses we surgically implanted pressure transducers into the cranial subarachnoid space of *Alligator mississippiensis* and analyzed the CSF pressure traces in both the frequency and temporal domains.

## Methods

### Live animals

Twelve live sub-adult (158–192 cm total length) American alligators (*Alligator mississippiensis*) were obtained from the Louisiana Department of Wildlife and Fisheries. The animals were housed communally in a 29 m^2^ facility that featured three submerging ponds, natural light, and artificial lights on a 12:12 cycle. The facility was maintained at 30–33 ℃; warm water rain showers were provided every 20 min, which helped maintain the facility at > 75% relative humidity. The alligators were maintained on a diet of previously frozen adult rats.

### Experimental preparation

When an individual animal was removed from the enclosure it was caught by noosing, its jaws taped shut around a bite pad using vinyl tape, and its fore and hind limbs taped in a retracted position. The alligator was then placed on a stiff board (244 × 28 × 3.8 cm thick), which exceeded the maximum width and length of the alligators used for this study. Six 2.5 cm wide heavy duty straps (Northwest Tarp and Canvas; Bellingham, WA) were used to secure the alligator to the board; the straps were tight enough to minimize movement of the animal but not tight enough to impede ventilation or circulation.

With the alligator’s mouth held open by the bite pad, a laryngoscope was used to depress the gular valve and expose the glottis. A cuffed endotracheal tube was inserted into the larynx and connected to a custom anesthesia system that included a ventilator pump (Harvard), Vaporstick anesthesia machine (Surgivet), isoflurane vaporizer (Surgivet), and Capnomac Ultima respiratory gas monitor (Datex-Engstrom). The alligators were maintained on a steady ventilatory pattern of 6–10 breaths per minute (depending on size) each with a tidal volume of 500 ml. Anesthetic induction was accomplished using 5% isoflurane; once a surgical plane of anesthesia was established the animal was maintained on 2–3% isoflurane depending on the response of the individual alligator.

The alligator’s EKGs were recorded using two silver chloride surface cup electrodes (019-477200, GRASS, Natus Medical, Pleasanton, CA), coated with a layer of conducting gel (Signagel, Parker Laboratories, Fairfield, NJ) and placed on the ventral surface of the animal on either side of the heart. The electrodes were connected to a P511 preamplifier (GRASS). A stainless steel surgical burr was used to bore an approximately 4 mm diameter portal through the sagittal midline of the skull just caudal to the orbits. This allowed for direct exposure of the dura mater; a small incision in the dura was used to inset a segment of PE tubing into the subarachnoid space. The PE tubing was connected to a P23AA fluid pressure transducer (Statham), both of which were filled with reptilian Ringer’s solution. The pressure transducer was mounted to the board at a fixed site immediately adjacent to, and level with, the alligator’s head, so that rotation of the alligator did not produce a pressure head between the PE tubing and the transducer. The implantation of the PE tubing was snug enough that no CSF leakage was observed, yet the functionality of the coupling was evident by the (pressure-driven) movement of the CSF along a distance of the PE tubing. The pressure transducer was coupled to a P122 preamplifier (GRASS). In five of the alligators a second surgical exposure was made to the vertebral column between the forelimbs. The exposure was deep enough to enable the implantation of a second PE catheter into the subdural space surrounding the spinal cord; this second implantation site was approximately 12 cm caudal to the implantation site in the skull. The second pressure catheter was connected to a second P23AA pressure transducer and PI22 preamplifier.

In seven of the alligators an additional surgical window was used to expose the suboccipital muscles on one side of the vertebral column; this exposure was roughly equidistant (rostral-caudal) between the two implanted CSF pressure catheters. A hands-free stimulating probe was positioned on the surface of the suboccipital muscles and stimuli provided by a S88 stimulator (GRASS). The CSF pressure, EKG, ventilatory pattern (from the exhalatory gas analyzer), and synced output from the muscle stimulator were recorded simultaneously (at 5.0 kHz) using the MiDas data acquisition system (Xcitex). The CSF pressure transducers were individually calibrated following each experiment.

### Influences on CSF pressure

The board holding the alligators was anchored to a rotating spindle machined to have, in addition to a stable horizontal stop, fixed “stops” at 15°, 30°, and 45° above and below the horizon. The animals were subjected to 60 or 90 s rotation trials, with the direction and magnitude of the rotation determined by die roll. The alligators were maintained in the rotated posture for 30 s; to avoid artifacts (vibration in the board) and transitions, only the last 10 s of the postural traces were analyzed. By adjusting the ventilator pump, three different ventilatory patterns were established: (1) resting (6–8 breaths per minute); (2) rapid (3 × the resting rate); and (3) apnea (0 breaths per minute). The alligators were given at least 90 s (at the resting rate of ventilation) between the ventilator trials. Twitch (10 ms, 3 V) and train (10 ms, 3 V, 60 pps) stimuli were presented to the suboccipital muscles using a hands-free stimulating probe. Visual contractions of the muscles were observed during each trial. Muscle stimulations were only performed on 6 of the *A. mississippiensis*. While the animals were still anesthetized with isoflourane, 0.1 mg/kg of epinephrine was administered (IM) into the triceps.

### Data analysis

**Fig. 1:**
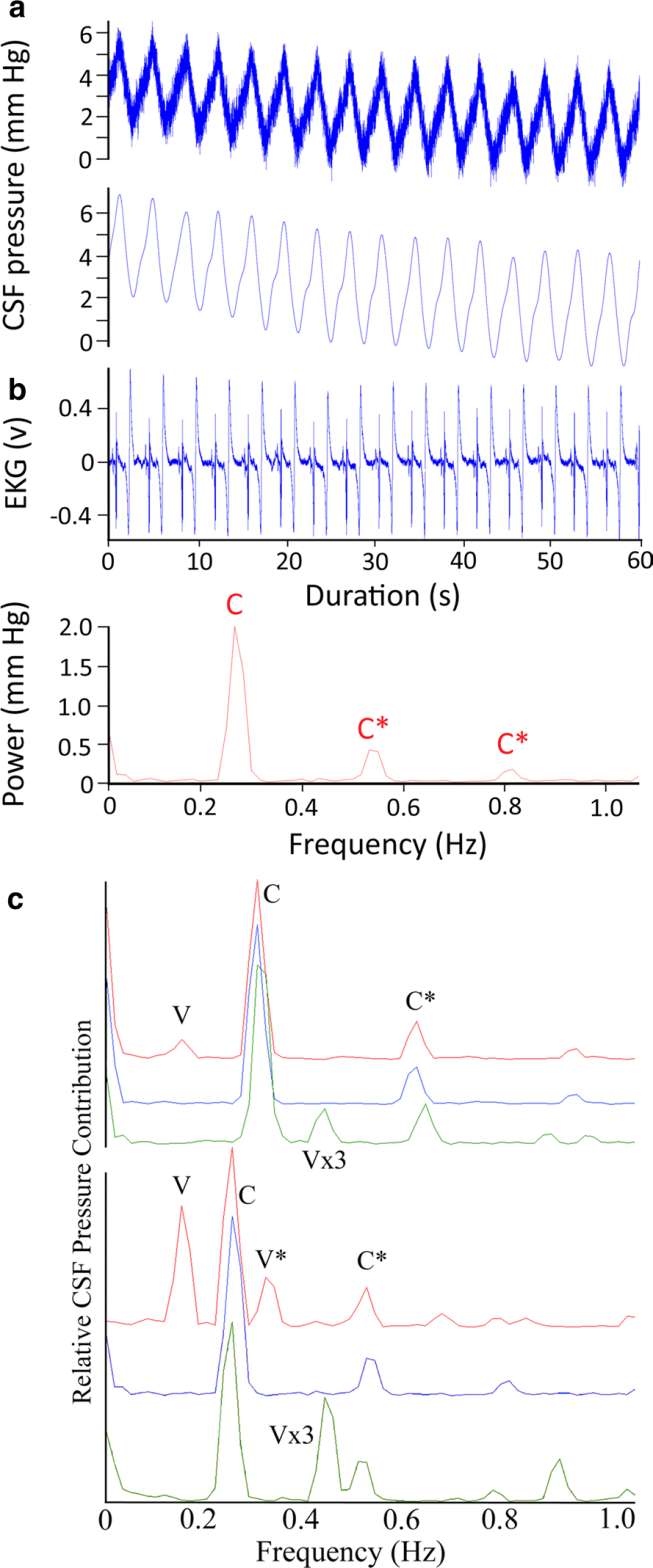
a 60 s data record of *Alligator mississippiensis* in apnea. The raw CSF and EKG tracings were recorded simultaneously, and show a temporal coupling between the cardiac cycle and CSF pulsations. The filtered (lower) CSF trace shows the relative stability of the pulsation amplitude (AMP), temporal pattern, and general shape. **b** Power spectrum analysis of the CSF trace shown in (**a**) reveals a fundamental spike (**c**) at the frequency of the heart rate (16 bpm), as well as two harmonics (C*); as expected during apnea, no ventilatory component was found in the power spectral analysis. **c** Composite Power Spectral analyses of CSF pressure waves recorded from two different alligators, both in a horizontal posture. For each alligator three (raw, unfiltered) CSF pressure traces were analyzed; at a resting level of 10 breaths per minute (red waves); during apnea or 0 breaths per minute (blue waves); and at a ventilatory rate of 3 × resting or approx. 30 breaths per minute (green wave). Both the spectral frequency and the overlap of the frequency spikes permit identification of the ventilatory (V) and cardiac (C) fundamental contributions, as well any harmonics (*)

While anesthetized with isoflurane the alligators typically had heart rates between 15 and 30 beats per minute (bpm), which roughly corresponded (but see below) to the CSF pulsation rate (Fig. 1a). The recorded signals were exported into SigView (SignaLab) for analysis. For temporal analysis the raw pressure trace was filtered using a 2 Hz low pass filter (Fig. 1a). Frequency analysis of the raw pressure trace involved FFT power spectral analyses (Fig. 1b) Since the cardiac (from the EKG) and ventilatory (from gas analyzer) rates were known, and three different ventilatory rates were used, the fundamental and harmonic spikes on the power spectrum could be identified (Fig. 1c) and used to determine the relative and absolute contributions to CSF pulsation amplitude.

### Spinal CSF flow

The cranial pressure catheter was disconnected from the transducer and used to introduce into the CSF a suspension of approximately 2.0 × 10^9^ artificial (sulfur hexafluoride) microspheres in 5 ml of saline (Lumason; Bracco Diagnostics, Monroe Township, NJ, USA). Doppler ultrasonographic records of CSF flow were quantified using an ultrasonography machine (Mindray M7; Nanshan, Shenzhen, P.R. China). A linear array probe (Mindary L12-4) was placed contralateral to the exposed suboccipital muscles and pulsed-wave Doppler ultrasonography used to detect the spread of the introduced microspheres in the CSF. The recordings presented were all taken prior to stimulation of the myodural bridge, and thus represent the resting or baseline flow condition. CSF flow velocity was quantified relative to the internally calibrated zero baseline of the ultrasonography machine. Data were collected from five of the alligators.

## Results

### Anatomy of the CNS in *Alligator*

**Fig. 2:**
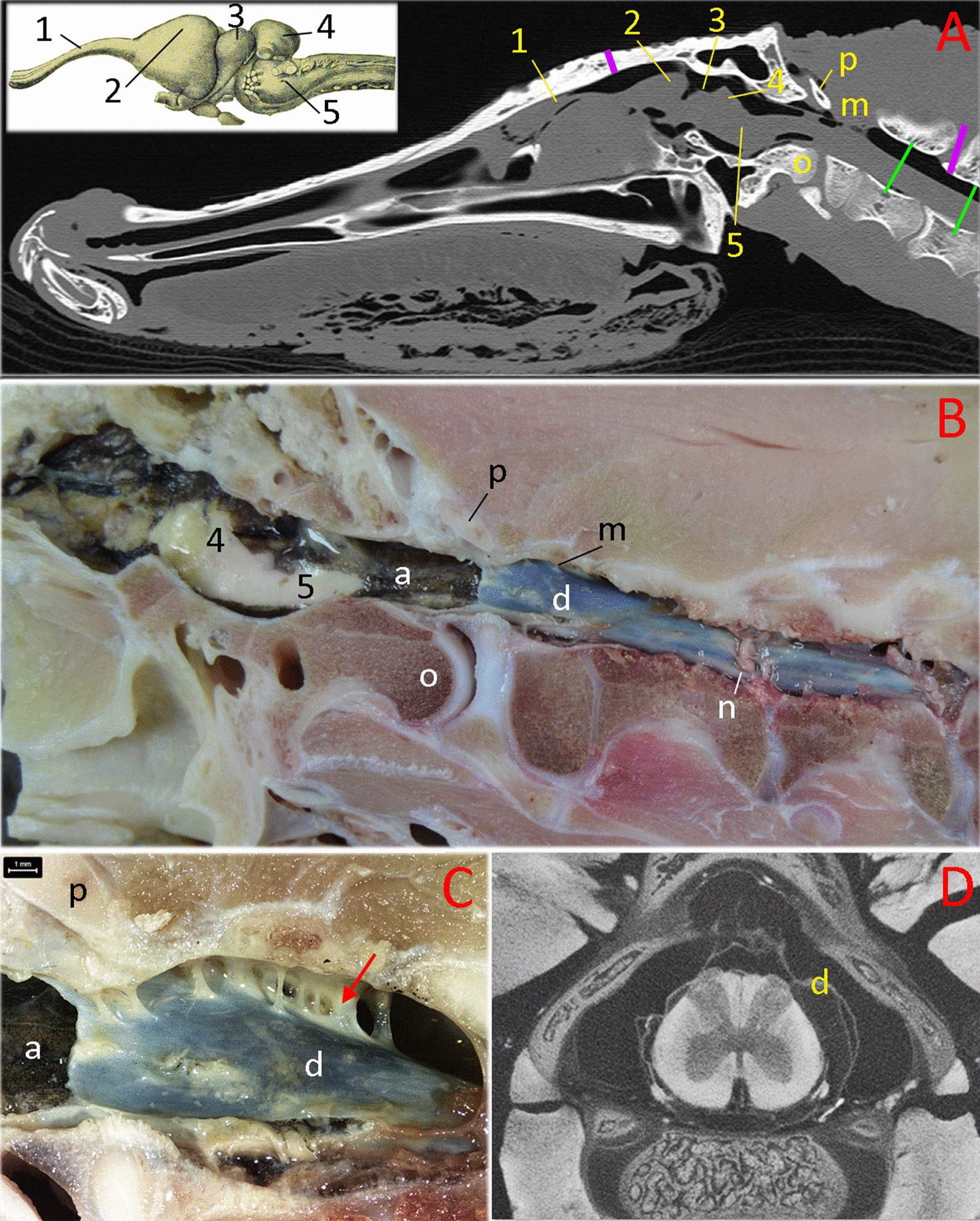
Morphology of the brain and spinal cord in *Alligator mississippiensis*. The specimens used for images A-C were sub-adult animals used in the present study. **a** parasagittal MRI, **b** photograph of a hemisected specimen, **c** magnified view of the myodural bridge (red arrow), **d** micro-CT transverse image through the cervical spinal canal of a hatchling specimen. The green lines in (**a**) indicate the region studied by Doppler ultrasonography in this study. The vertical purple lines indicate the surgical openings made to record CSF pressure; the marker on the skull is accurate. The actual surgical site to record spinal CSF pressure was nine vertebrae more caudal. Abbreviations: 1—olfactory tract; 2—cerebral cortex; 3—optic lobe of the midbrain; 4—cerebellum; 5—medulla oblongata; a—arachnoid mater; d — dura mater; m—myodural bridge; n—spinal nerve; o—occipital condyle; p—proatlas

There are some key features of *Alligator* nervous system which may be particularly relevant as the reader puts the experimental data into context. The brain and spinal cord of *Alligator* are essentially linear, lacking the prominent flexures found in mammals (Fig. 2). In *Alligator* the cerebellum is a domed structure, which houses a large branch of the 4th ventricle, that rests relatively further rostral to the foramen magnum than the condition in humans or other mammals (Fig. 2). There is a prominent myodural bridge in *Alligator* formed by a direct insertion of the sub-occipital muscles onto the dural sheath (Fig. 2c). The spinal epidural space of *Alligator* is devoid of the prominent adipose tissue found in mammals; there is a dorsal spinal vein, but the remainder of the epidural space is primarily empty (Fig. 2d). The dura mater of *Alligator* is a bluish-white sheath, while the arachnoid is heavily pigmented (Figs. 2b, c); the "attachment" between these two meningeal layers do not appear to be as robust as it is in mammals.

### CSF pressure wave

**Fig. 3:**
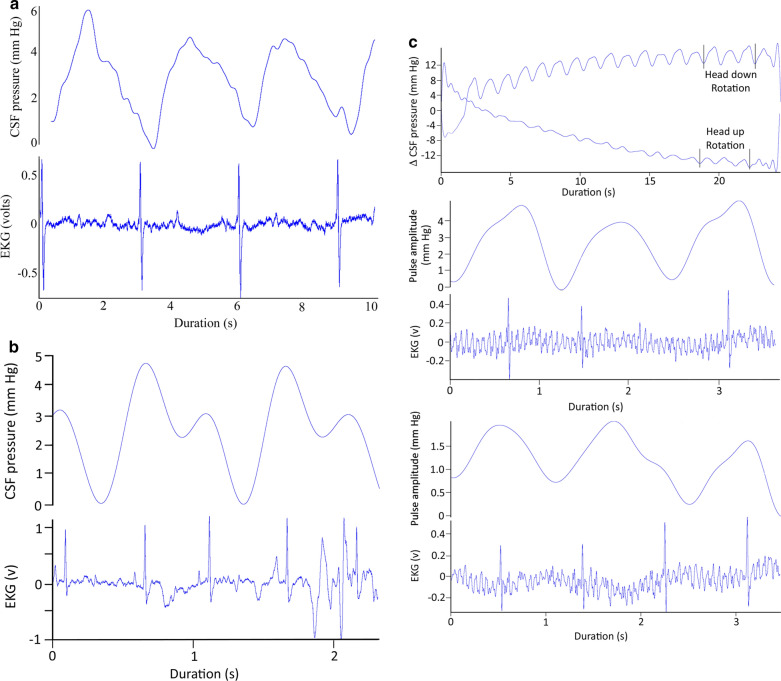
**a** Simultaneous recordings of CSF pressure wave (upper trace) and EKG (lower trace) from an anesthetized, horizontal, *Alligator mississippiensis*. This 10 s segment is a portion of a 30 s trial pulled out to represent the size, shape, and temporal patterns of "typical" CSF pulsations in *Alligator*. **b** Simultaneous recordings of CSF pressure wave (upper trace) and EKG (lower trace) from an anesthetized, horizontal, *A. mississippiensis* exhibiting tachycardia. Note that compared to CSF pulsations at lower heart rates (**a**), these CSF pulses occur sooner after the R peak, are shorter in duration, and appear to "piggy back" on the diastolic slope of earlier pulses. **c** Influence of orthostatic gradient on CSF pulsations in *A. mississippiensis*. Upper traces are CSF pressures recorded during two 15° rotations in opposite directions; note that both traces start at 0 on the Y axis. Two series of terminal pulsations were taken from the end of the trials (indicated by the black vertical lines). The CSF pulsations and simultaneous EKG recordings from the head down rotation are shown in the middle of the figure, those from the head up rotation are shown in the bottom of the figure. Note the different scales used for the CSF pulsations from the head up and head down rotations

While the alligator was in a horizontal position, the CSF pressure traces were always pulsatile. There was variation in the size and shape of the CSF pressure pulses. The CSF pulsations often include a poorly demarcated "shoulder" on the systolic side or a "peak" on the dialostic side, but these were frequently missing (Figs. 1a, 3a). A “typical” trace is illustrated in Fig. 3a; this alligator was horizontal, had a heart rate of 20 bpm, and was ventilating at 8 bpm. In this 30 s record the cardiac cycle (measured as the R-R interval) had a mean of 3.07 s (s.d. = 0.006). The systolic (increasing) side of the CSF pulsations began 0.43 s (s.d. = 0.04), or 14% of the cardiac cycle, after the R peak of the EKG, and peaked at 1.5 s (s.d. = 0.05) or 49% of the cardiac cycle after the R peak. The CSF pulsations were asymmetric, with the systolic side lasting 1.08 s (s.d. = 0.06) or 35% of the cardiac cycle, while the diastolic side lasted 1.99 s (s.d. = 0.05) or 65% of the cardiac cycle (Fig. 3a).

The 12 alligators examined for this study had a mean intracranial pressure (ICP) of 4.6 mm Hg (s.d. = 0.44). The CSF pressure pulses had a mean amplitude (or AMP) of 4.58 mm Hg (s.d. = 0.46); the amplitudes of the pulses were more variable than the durations. Human studies have often described the CSF pulsations by dividing the pulsatile pressure by the duration to the pressure peak (the p/t value, [31]); in this record, the mean p/t value would be 4.25 mm Hg/s (s.d. = 0.46). All of these parameters varied among and between the alligators.

### Variations in the CSF pulsatility

If the alligator's heart rate increased the CSF pulsation curve would shorten. Before the alligator exhibited tachycardia, defined for this study as a heart rate above 30 bpm, the shape of the pulsation curve would change (Fig. 3b). The systolic side of the curve begins sooner after the R peak of the EKG (mean of 0.24 s in the trace in Fig. 3b), and the pulsation itself is much shorter (1 s for the complete "pulse" in Fig. 3b). A one second duration appears to be near the minimum duration for the CSF pulsation curve in *Alligator*; as the heart rate increased above 30 bpm, what appear to be secondary pulses would appear on the diastolic side of the CSF pulsation curve (Fig. 3b). When the animal was horizontal these secondary pulses had a lower amplitude than the main pulse. If a tachycardic alligator was tilted head down, the amplitude of the secondary pulse increased while the main pulse remained relatively constant.

Orthostatic gradients were created by rotating the alligator into a head up or head down posture. Rotating the alligators did not induce a change in heart rate (Fig. 3c); however, it did alter the temporal relationship between the cardiac cycle and the CSF pulsations. The regular temporal relationship between the R peak of the EKG and the onset of the systolic side of the CSF pulsation (Figs. 1a, 3a, b) disappeared once the alligator was rotated in either direction (Fig. 3c). The AMP also changed; with each degree of rotation the AMP increased by roughly 0.1 mm Hg during head down rotations, and decreased by 0.1 mm Hg during head up rotations. The data presented in Fig. 3c are from an alligator with relatively low AMP (3.25 mm Hg) while resting; the AMP decreased to 1.7 mm Hg during 15° head down rotations (bottom trace of Fig. 3c), and increased to nearly 5 mm Hg during 15° head up rotations (middle trace of Fig. 3c). Though the amplitude (AMP) of the CSF pulsations change with rotation, the basic shape of the pulsation curve remains the same (Fig. 3c). Due to the rate of decrease in AMP during head up rotations, the CSF pressure tracings were almost always non-pulsatile during 45° head up rotations, and were commonly so during the 30° head up trials.

**Fig. 4:**
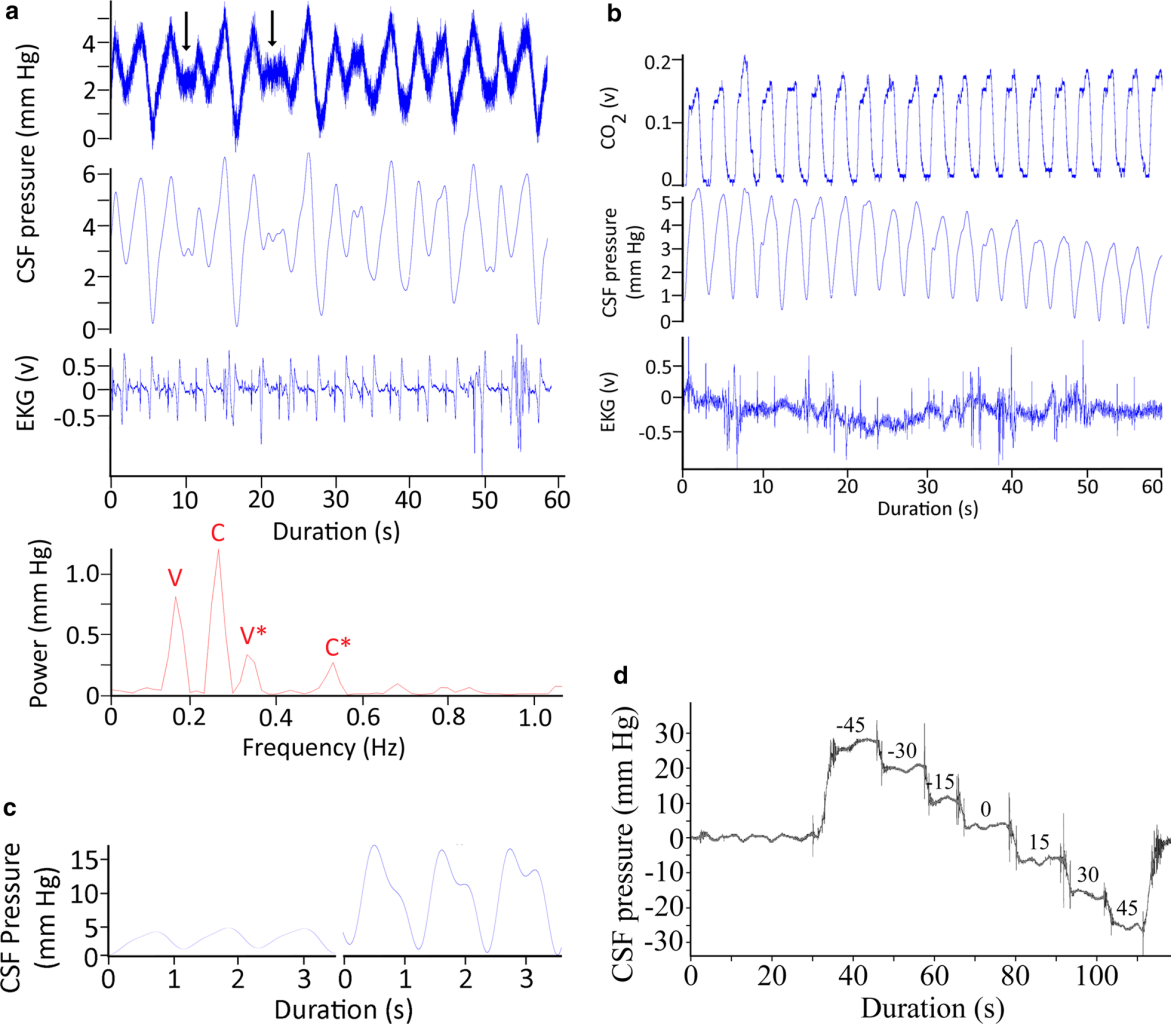
**a** 60 s data record of *Alligator mississippiensis* ventilating at 10 breaths per minute. The raw CSF and EKG tracings were recorded simultaneously. These traces were recorded shortly after those shown in Fig. 1a, here there are "disruptions" to the periodicity of the CSF pulsations (black vertical arrows). These disruptions, evident in the raw and filtered CSF traces, match the frequency of the ventilatory cycle, which was also evident in the power spectral analysis. C—heart rate, V—ventilatory rate, *—harmonics. **b** 60 s data record of *A. mississippiensis* showing simultaneously recorded CO_2_ consumption, CSF pressure, and EKG. Note the clear temporal correlation between the CSF pulsations and the ventilatory cycle, and the lack of a temporal pattern between the R peaks of the EKG and the CSF pulsations. **c** Two 3 s data tracings pulled from a 3 min continuous recording of CSF pressure in *A. mississippiensis*. The traces on the left were recorded shortly before the administration of epinephrine; those on the right were recorded approx. 40 s after the administration. **d** Influence of rotation of *A. mississippiensis* on CSF pressure. These rapid sequential rotations demonstrate the linear relationship between posture and CSF pressure

The ventilatory pattern influenced the nature of the CSF pulsations. Figure 1a presents a data record from an anesthetized horizontal alligator in which the ventilator attached to the cuffed tracheal tube had been turned off, forcing the alligator into a state of apnea. During this apnea the alligator had a heart rate of 16 bpm, these beats were synchronized to the 16 CSF pressure pulses recorded during this trial, and were evident in the power spectral analysis where the major contribution was found to be at 0.27 Hz (or 16/60, Fig. 1b). Within two minutes of the data trace shown in Fig. 1a the ventilator was turned back on, and the data trace shown in Fig. 4a was recorded. The alligator's heart rate was still 16 bpm, but CSF pulsations were more irregular, and the synchrony between the cardiac cycle and the CSF pulsations was reduced. In both the raw and filtered CSF pressure traces these disruptions were evident (black arrows in Fig. 4a), and the frequency of the disruptions matched the frequency of the ventilatory cycle, which was also evident in the power spectral analysis (Fig. 4a). The CSF pulsations that occurred during these ventilatory "disruptions" were characterized by a reduced AMP, and seemed to occur prior to the completion of the previous pulsation. In this way, these ventilatory pulsations resembled the unusual curves seen during tachycardia (Fig. 4b), though the AMP was greater and the heart rate was not tachycardic.

The timing, or synchrony, of the CSF pulsations included a third pattern which was less frequently encountered. In multiple trials with multiple alligators the CSF pulsations were temporally correlated not with the cardiac cycle, but with the ventilatory cycle (Fig. 4b). The CSF pulsations recorded during these trials had "typical" AMP and temporal features (Fig. 4b) but the temporal features bore no relationship to the cardiac cycle.

The administration of epinephrine into the triceps produced large spikes in the AMP (Fig. 4c). The CSF pulsation retained the typical shape (save for the exaggerate height) and duration; the epinephrine did not cause an increase in heart rate (Fig. 4c). Epinephrine's influence on the CSF typically did not manifest until around one minute after injection, and the resulting spike in CSF pressure only lasted approximately 45 s.

### Orthostatic gradients and CSF Pressure

**Fig. 5:**
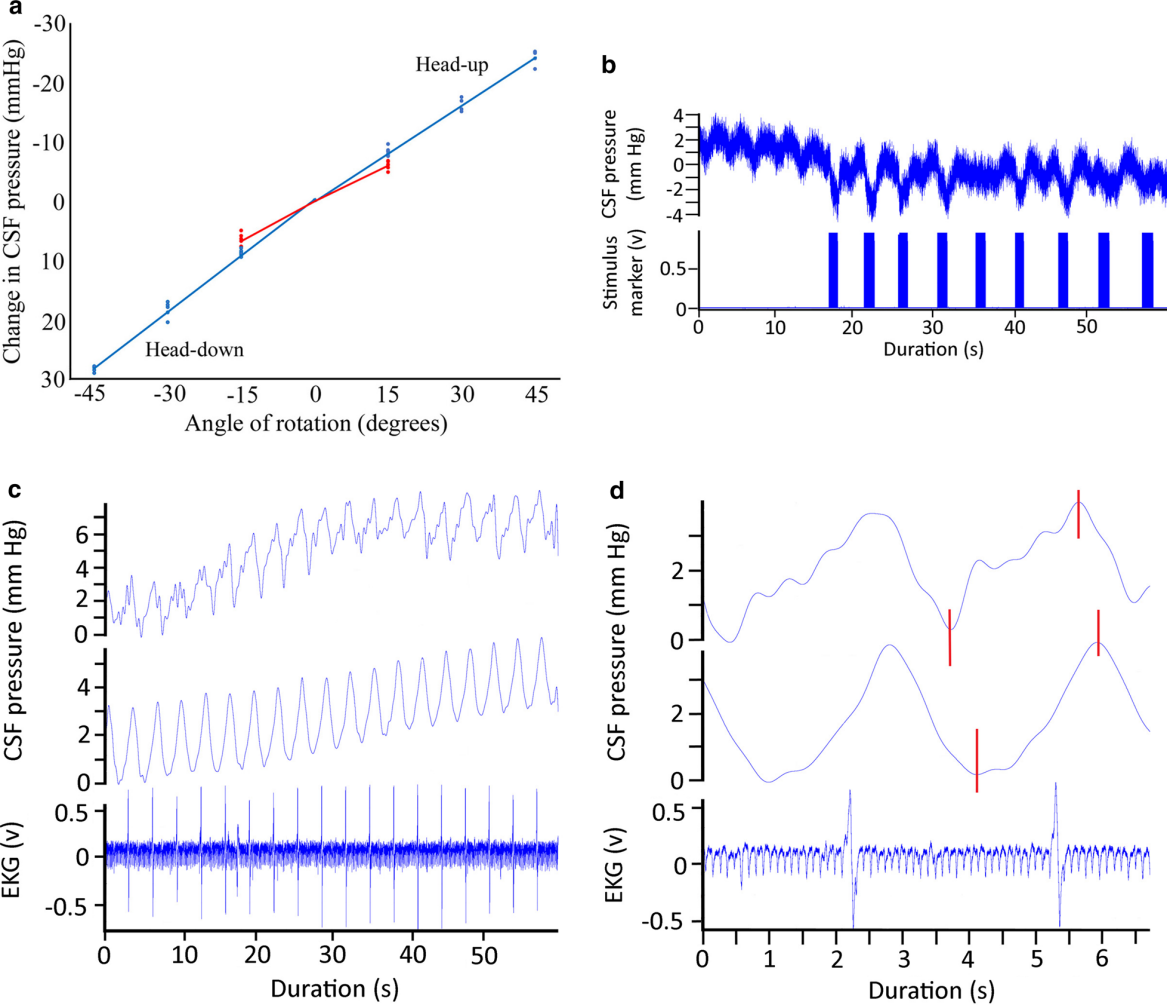
**a** Data from the cranial (blue) and spinal (red) implantation sites during rotations. Separate curve fitting was done for the head-up and head-down rotations, though the slopes were not significantly different. Rotations resulted in significantly lower changes in CSF pressure at the spinal recording site (red) compared to the cranial recording site (blue). **b** Raw simultaneous data recordings of CSF pressure and electrical stimulation of the suboccipital muscle in *Alligator mississippiensis*. Stimulation of the muscle was associated with a decrease in CSF pressure. Variation in this response may represent variation in stimulus strength (caused by slight displacement of the muscle relative to the stimulating probe) or possibly fatigue of the muscle. **c** Simultaneous recordings of CSF pressure from the cranium (top) and spinal canal (middle) of *A. mississippiensis*. These two CSF traces show clear synchrony, and are synchronized with the (simultaneously recorded) EKG trace (bottom). **d** Simultaneous recordings of EKG (bottom trace) as well as CSF pressure from the cranium (top) and spinal canal (middle) of *A. mississippiensis*. There is a temporal offset between the cranial and spinal CSF pulses (red vertical lines), such that the spinal pulse lags behind the cranial pulse by approximately 200 ms

Relatively rapid rotation of *Alligator mississippiensis* through a range of postures from 45° head up to 45° head down produces a linear response in CSF pressure (Fig. 4d). Slower rotations were performed to quantify the alligator’s response; after the initial change in CSF pressure, the CSF stabilized at the new pressure (Fig. 3c). Each degree of head-up rotation caused a decrease in cranial CSF pressure of 0.6 mm Hg; each degree of head-down rotation caused an increase in cranial CSF pressure of 0.6 mm Hg (Fig. 5a). The spinal CSF pressure was recorded closer to the alligator’s axis of rotation; paired t-tests demonstrate that the changes in CSF pressure were significantly lower at the spinal site than at the cranial site during both the head-up (t = 3.86, df = 5, *p* = 0.012) and head-down (t = 3.83, df = 5, *p* = 0.012) rotations (Fig. 5a). Over the course of the rotations performed for this study there was no evidence for any compensatory regulation of the CSF pressure.

### Myodural bridge and CSF pressure

Twitch or train stimuli applied to the suboccipital muscles resulted in corresponding decreases in CSF pressure (Fig. 5b). Twitch stimuli reduced the pressure by 0.5 mm Hg (s.d. = 0.17), while the train stimuli caused a 1.9 mm Hg decline in the CSF pressure (s.d. = 0.25). This experimental stimulaion of the suboccipital muscle did not produce CSF pulsations, but is best understood as an alteration of the mean CSF pressure and alteration of existing CSF pulses.

### Cranial and Spinal CSF pulsations

**Fig. 6:**
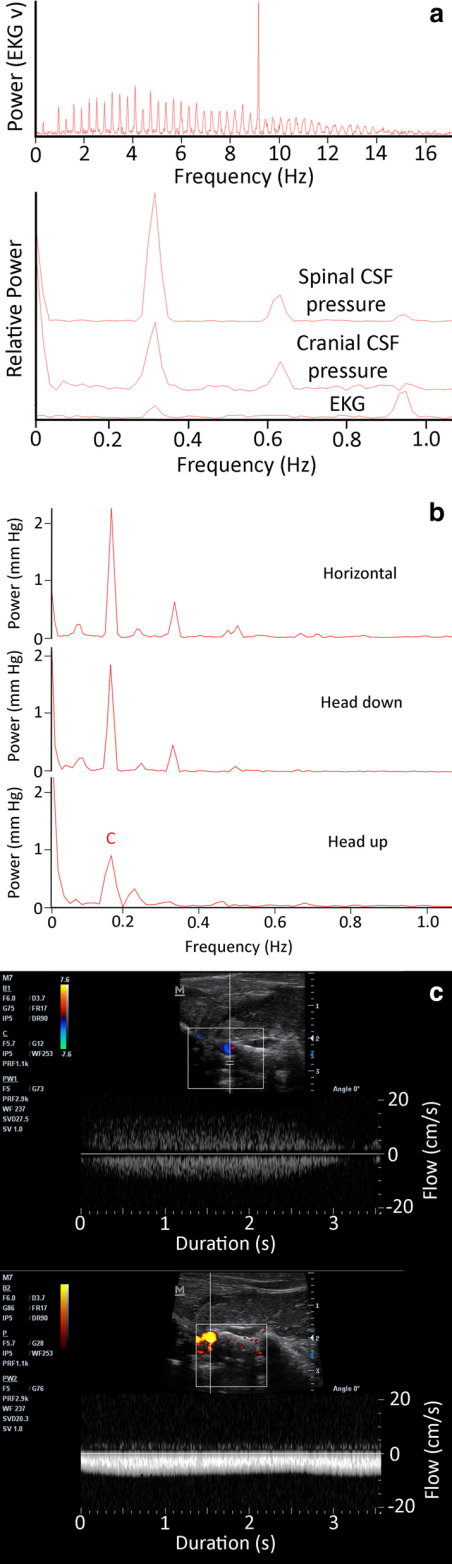
**a** Power spectral analyses of simultaneously recorded EKG, cranial CSF pressure, and spinal CSF pressure waves from *Alligator mississippiensis*. The EKG traces are rich in harmonics (upper trace); this analysis was only concerned with the fundamental frequency and first two harmonics (which occur on the far left of the upper trace). The lower panel includes the same low frequency region of the EKG analysis, overlain with the power spectral analyses of the spinal and cranial CSF pulsations (these were placed on the same Y axis). The power of the CSF pulsations clearly captures the fundamental frequency of the cardiac cycle, as well as the first and second harmonic; however, the power of the fundamental frequency is lower in the recordings from the skull compared to those from the spinal canal. **b** Power spectral analyses of CSF pressure traces from the same *Alligator mississippiensis* during a period of approximately 10 min. During this time recording were taken from the animal in a horizontal (resting) posture, it was rotated to 15° degrees head up, returned to horizontal for 3 min, then rotated to 15° head down. Only CSF pressure data from the terminal (stable) portion of each rotation was analyzed. C—heart rate. **c** Doppler ultrasonographic traces of the microsphere-labelled CSF in the spinal subarachnoid space of two different Alligator mississippiensis. Note that while the two traces differ in flow velocity (the Y-axis), the have a similar ~ 0.3 Hz pulsatile frequency, and a similar unidirectional flow profile. In both traces the CSF flow is indicated below the baseline of the graph (meaning the CSF was flowing toward the head); the signal above the baseline is an aliasing of the Doppler trace

The subarachnoid space is continuous between the spinal canal and the skull (Fig. 2), so it is not surprising that CSF pressures recorded at the two sites (12 cm apart) showed clear synchrony (Fig. 5c). A more detailed examination reveals that there is a temporal offset between the two CSF pressure pulses. Quantifying the offset from 20 traces evenly divided among the 5 alligators revealed a mean offset of 196.7 ms (s.d. = 9.9), with the pulse in the spinal canal being delayed compared to the pulse in the skull (Fig. 5d). Simultaneous cranial and spinal CSF pressure recordings were made during periods of apnea, to remove any complications from the ventilatory cycle. These apnic periods had similar temporal offsets (means = 193.7 ms, s.d. = 8.9). Power spectral analysis (Fig. 6a) reveals that the power of the fundamental frequency corresponding to the cardiac cycle is lower in the CSF pulsations recorded from the skull compared to those from the spinal canal (paired t-test; t = 9.12, *p* = 0.0004, n = 5).

### Cardiac and ventilatory contributions

The FFT and power spectral analyses were performed on all 12 alligators and the height (power) of the fundamental frequencies of the ventilatory and cardiac contributions compared. The relative contribution of the cardiac cycle to CSF pressure was on average 3.6 times larger than that of the ventilatory cycle. Interestingly, this mean of 3.6 × was associated with a s.d. of 4.2; the standard deviation was greater than the mean because in some of the records analyzed the relative contribution of the ventilatory cycle was greater than that of the cardiac cycle. The analysis of relative contributions only entailed the fundamental frequencies. A second analysis of absolute contributions was performed; in this analysis the power of each harmonic was added to that of the fundamental frequency. In terms of absolute contribution, the cardiac cycle accounted for 1.7 mm Hg of the CSF pulsation AMP (s.d. = 0.83) while the ventilatory cycle accounted for 1.1 (s.d. = 0.93). The absolute contributions of the cardiac and ventilatory cycles were more similar than their relative contributions because the power spectral analysis more commonly revealed harmonics attributable to the ventilatory than the cardiac cycle.

The relative and absolute contributions of the cardiac and ventilatory cycles to the CSF pulsations were not static. If ventilatory and heart rates were stable, but the alligator was rotated into a head up or head down posture, shifts were observed in the power spectrum (Fig. 6b). These shifts were mainly in relative power, rather than in frequency; ANOVA of the absolute power contributions from the 12 alligators revealed significant (F = 602.9, *p* = 1.11 × 10^–16^) differences between the three postures. Tukey's Post = hoc analysis revealed no significant difference between horizontal and head-down contributions (*p* = 0.311); but significant differences between the head-up posture and both the horizontal and head-down postures (for both comparisons, *p* = 0.001).

### Spinal CSF flow

The CSF flow velocities around the cervical spinal cord were characterized by long-duration pulsatile flow (Fig. 6c). The 10 CSF flow velocities quantified had a mean pulse duration of 2.9 s (s.d. = 0.2). The velocity of the CSF ranged between 4 and 9 cm/s, and was fairly consistent during the flow pulse. This means that in some of the alligators, an individual CSF pulsation was displacing the CSF over 25 cm. These CSF flow velocities were consistently recorded from the dorsal surface of the cervical spinal cord. In this region only cephalic flow was recorded; the flow would decrease to 0 cm/s between the pulses (Fig. 6c), but did not reverse to caudad flow in any of the alligators.

## Discussion

The changes in CSF pressure that occur during rotation (Figs. 4d, 5a) are similar to expectations; assuming a 50 cm distance from the axes of rotation to the pressure cannula, a 45° rotation would produce a 27 mmHg pressure head. Similar postural/barostatic shifts in intracranial pressure have been documented in mammals [32]. Alligators do not exhibit a compensatory baroreflex when orthostatically challenged [29]; head-up rotations cause a significant reduction in carotid blood flow in *A*. *mississippiensis*, while head-down rotations are associated with a significant increase in carotid blood flow [30]. A lack of compensatory baroreflex, and significant alterations of blood flow patterns due to gravitational gradients, have been previously described in reptiles [33, 34]. These postural shifts in the blood distribution of *A. mississippiensis* would cause a corresponding change in cardiac output and cranial artery/arteriole pulsation amplitude, and would presumably shift the cranial compliance [35, 36], which could explain the differences in CSF pulsation amplitude observed during rotation (Fig. 3c).

Pulsations have been reported in the extra brain fluid of the Port Jackson shark (*Heterodontus portusjacksoni*) [37], but there are few additional descriptions of CSF pulsations from non-mammals. CSF pressures and pulsations have been quantified from a variety of mammalian species, including: cats [38]; rabbits [39]; dogs [40]; monkeys [41]; horses [42]; mice [43]; and humans [44]. These mammalian studies report similar CSF pressures, pulsations with similar temporal relationships to the cardiac cycle, and frequency analyses which consistently show that the cardiac cycle has the greatest contribution to CSF pressure pulsation [45].

In *Alligator* the CSF pulsations do not show a consistent temporal relationship to the cardiac cycle, even within an individual and while the animal is horizontal (compare Figs. 1a, 4a, b and 5d). As in mammals, the cranial arteries course along the base of the alligator brain [46, 47]. Blood flow through these vessels may be more variable in *Alligator* than in a mammal, owing in part to the multiple shunts and physiological regulations of cardiac outflow in *Alligator* [48–50]; but these are primarily voluntary systems which would have been impeded, if not eliminated, by the anesthesia. If the CSF pulsations in *Alligator* are driven by arterial pulsation of some form, be it pulsations of the large arteries surrounding the brain [51], arteriole driven piston-like pulsation of the brain within the dural sac [52], or pulsations of the choroid plexus [53], how can there be such temporal variation between the cardiac cycle and the CSF pulsations?

Ventilatory movements of the mammalian thorax often produce synchronous CSF pressure pulsations [54, 55], though these pulsations are of a lower amplitude than those attributed to the cardiac cycle [45]. The mechanics of these ventilatory pulsations are uncertain; direct expansion of the thoracic spinal dura has been hypothesized [56] as has pressure influences on the cephalic venous drainage [57]. Though alligators share with mammals cyclic changes in thoracic pressure associated with ventilation [58], In some ways *Alligator* ventilation is different from that of mammals; alligators are intermittent breathers [24], and can use a hepatic pump or abdominal displacement to drive ventilation [25, 59]. A recent study [60] argued that during the evolution of tetrapods when axial musculature de-coupled from locomotion to drive ventilation, it also produced a novel propulsive mechanism for the CSF. Alligators were used for this study specifically because their unusual plasticity in ventilatory mechanics suggests that the functional link between ventilation and CSF propulsion would not be as developed in this group of tetrapods.

In the experiments reported herein, the alligators were actively ventilated at a frequency and tidal volume similar to what has been reported from non-anesthetized alligators [59, 61]. Although during these experiments the alligators were undergoing cyclic changes in intrathoracic pressure (like a ventilating mammal), the relationship between ventilation and CSF pulsations in the alligator is unlike anything every reported from a mammal. In some cases the CSF pulsations appear to be driven solely by the ventilatory cycle (Fig. 4b), whereas in others the ventilatory cycle appeared to interfere with the CSF pulsations (Fig. 4c). The unique relationship among the cardiac, ventilatory, and CSF pulsatory cycles of *Alligator* is also apparent during frequency analysis. Unlike the situation in mammals, in which the cardiac cycle makes the greatest relative and absolute contribution to CSF pulsations [45], in *Alligator* there was more variation (Fig. 1c) and sequences were analyzed in which the ventilatory component had a greater relative and absolute contribution than the cardiac component.

Isoflurane causes respiratory and cardiac depression in reptiles, presumably through increasing vagal tone [62]. Reptiles appear to have the same autonomic control over cardiorespiratory interactions as mammals [63]. The increased vagal tone produced by the isoflurane disrupted some of these controls: though three different ventilatory rates (apnea, 8 breaths per minute, and 24 breaths per minute) were applied to *A. mississippiensis*, there were no corresponding changes in heart rate (Fig. 1c). Previous workers have argued that ventilatory movements were transferred to the CSF by intrathoracic pressure changing venous drainage such that the venous pressure of the dural sinuses would change [64]. An increase in intracranial venous pressure would lead to an increase in intracranial CSF pressure, but would not disrupt the CSF pulsations; rotating the alligators head down caused the venous blood to pool in the head [30], and was associated with an increase in CSF pressure (Figs. 4d, 5a) but also resulted in an increase in CSF pulsation amplitude (Fig. 3c). Frequency analysis demonstrated that when ventilatory movements were held constant, but the alligator was rotated head-up (so arterial pulsations were reduced) the relative contribution of the cardiac peak within the CSF pulsation was decreased while the ventilatory peaks were increased (Fig. 6b).

Accordingly, we follow [56] and hypothesize that in *Alligator mississippiensis* ventilatory movements result in displacement of the spinal dura adequate to create CSF pulsations. The capacity of the spinal dura to create these pulsations is demonstrated by the activation of the myodural bridge (Fig. 5b). The myodural bridge only has a contact area of approximately 3.5 cm with the spinal dura (and thus is a small fraction of the thoracic length), yet twitch stimulation causes a significant decrease in CSF pressure even though the dura was estimated to be displaced only 0.1 mm [60]. We do not propose that the myodural bridge contracts rhythmically, only that the influence of the myodural bridge on CSF pressure suggests that a rhythmic thoracic dural displacement could well produce the type of CSF pulsations recorded in *Alligator mississippiensis*.

The relationship between between the ventilatory cycle and CSF pulsations in the alligator may also relate to the unusual long duration of the CSF pulsations reported in this study. The CSF pulses in *A. mississippiensis* had a frequency of approximately 0.3 Hz (Figs. 1b, 3a), which is far lower than the ~ 1.3 Hz of human CSF pulsations [45, 65], but reflects the slow heart rate of *Alligator* when anesthetized with isoflurane [66]. In mammals as heart rate increases there are corresponding increases in CSF pulsation frequency and decreases in CSF pulse duration; in mice, where the resting heart rate is approximately 600 bpm, [43] recorded CSF pulsations with a frequency of around 6 Hz, some 20 × that of the alligator. In the alligator an increase in heart rate increases the frequency of the pulsations, but the duration of each CSF pulsation seems to hit a minimum at around 1 s. This can lead to CSF pulsations originating from the diastolic slope of earlier pulsations (Fig. 3b). These summated curves precluded quantifying the duration of these CSF pulsations. We are treating 1.0 s as the approximate minimum duration of a CSF pulsation in *Alligator* because we see discrete pulsation of slightly greater than 1.0 s (Fig. 3c) but did not record any discrete pulsations with durations less than 1.0 s. This minimum duration is very close to the inhalatory duration of the ventilatory cycle (Fig. 4b), which is presumably when the spinal dura would be displaced and a CSF pulsation produced. This hypothesis that in *Alligator* the ventilatory contributions create a minimum duration to the CSF pulsations is supported by the observation that only a portion of "summated" CSF curves (Fig. 4b) increase in amplitude during head-down rotations. Nevertheless, the presence of an (apparent) minimum duration to the CSF pulsations, even during apnea, indicates that other factors are influencing the variation in CSF pulse duration.

Studies of the bulk flow of CSF between the cranial and spinal sub-arachnoid spaces of mammals have demonstrated pulsatile flow, but the pulsations are quite different to those reported herein from *Alligator*. In humans, and other mammals, these CSF flow pulses are of much shorter duration, typically one second or less [67], compared to the 3 s duration in alligators (Fig. 6c). Furthermore, the mammalian flow pulses are typically bi-directional [68], though asymmetry in the two flow directions may yield net directional flow [69]. The flow velocities recorded in *Alligator* were unidirectional; though the CSF slowed down at the end of the pulse it did not change direction. These *Alligator* flow velocities were recorded from the subarachnoid space dorsal to the spinal cord; there is no evidence that all of the spinal CSF fluid was flowing unidirectionally. Similar "large scale" unidirectional CSF flow pulses have been reported from zebra fish embryos [70, 71] and previous studies on human CSF flow have wrestled with flow velocities and patterns that seem "discordant" with potential reservoir volumes [72].

Spectral frequency analysis (Figs. 1c, 6b) was used to segregate the cardiac and ventilatory components of the CSF pulsation, but the resulting power spectra were consistently different from those reported from mammalian studies. Power spectral analyses of mammalian CSF pulsations reveal a large number of higher-order harmonics [45, 65], which have been interpreted in terms of skull resonance and system compliance [45, 65, 73, 74]. In this analysis of *Alligator*, spectral peaks above a 3rd order harmonic were never recovered. As the spectral analysis of the EKG waveform shows (Fig. 6a), this was not due to an inherent limitation of the recording/analysis protocol, but seems to reflect the nature of the CSF pulsations themselves. Setting aside the significance of these low energy components of the pulsation, we hypothesize that their absence may reflect the nature of the reptilian meninges. Unlike the situation in mammals, in *Alligator* the dura is not fused to the skull [75], and there appears to be more mobility between the meningeal layers. As such the meninges in *Alligator* may be more compliant than those of mammals, which could effectively dampen some of the CSF pulsation.

Perhaps the most intriguing finding to come out of this study is that the CSF pulsations "differ" between the skull and spinal canal, despite the continuity of the CSF and subarachnoid spaces. When the "same" CSF pulsations are recorded from the cranial and spinal CSF there is a clear synchrony (Fig. 5c). Spectral analysis reveals a slightly different profile between the two, perhaps indicative of the large venous sinus in the epidural space of the spinal canal [76]. The differences between the cranial and spinal CSF pressure curves could be a manifestation of compliance [77, 78] or reflexional differences; similar differences have been previously reported from human studies [79]. Closer examination shows that the CSF pulsations have a temporal offset of approximately 200 ms, with the spinal pulses lagging behind the cranial pulses. This temporal offset means there is also a pressure offset (of up to 1.5 mm Hg); if the curves are artificially synchronized the pressure difference is less than 0.5 mm Hg. The cranial and spinal pressure transducers were only 12 cm apart; sound wave propagating through fluid would cover 12 cm in roughly 0.08 ms. An earlier study comparing intracranial and lumbar pressure recordings in humans [80] also reported a temporal offset greater than sound wave propagation, and noted that the delay was decreased at higher pressures, presumably due to changes in compliance. Similar temporal offsets between cranial and spinal CSF pulsations have been found in humans [81], suggesting that there is a real, previously unexplained, delay or temporal shift that occurs as part of the CSF transition from the skull to the spinal canal.

## Conclusions

All vertebrates have CSF systems involving pulsatile and net flow influenced by cardiac, respiratory, vasomotion and orthostatic forces the relative importance of which are currently debated, particularly in light of the CSF´s role in brain clearance. In *Alligator mississippiensis* CSF pulsations have amplitudes of approximately 5 mmHg and durations around 3 s (Figs. 1a, 3a), and the bulk flow of CSF around the spinal cord includes clear unidirectional flow that is maintained for a similar duration (Fig. 6c). Furthermore, the respiratory pressure gradients can be of both longer duration and larger in amplitude than the cardiac-related pressures (Figs. 1c, 3b), and the separation between pulsatile and bulk CSF flow may be diminished or eliminated. Only further comparative studies will demonstrate if the large influence of ventilation on the CSF, and the long duration CSF pulsations and CSF flow profiles are a unique attribute of *A. mississippiensis*, or a shared reptilian characteristic. The current study demonstrated that all of the cardiac, respiratory, vasomotion and orthostatic forces are significant players of CSF dynamics in alligators, and that the link between the pulsatile and bulk CSF flow regimens may be more amenable to experimental exploration and manipulation in this species.

## Data Availability

All reasonable requests for data will be gladly granted by the corresponding author.

## References

[1] Jones H (1979). Comparative aspects of the cerebrospinal fluid system in vertebrates. Sci Prog.

[2] Cserr H, Bundgaard M (1984). Blood-brain interfaces in vertebrates: a comparative approach. Amer J Physiol.

[3] El Sayed T, Mota A, Fraternali F, Ortiz M (2008). Biomechanics of traumatic brain injury. Comput Methods Appl Mech Engrg.

[4] Segal M (2001). Transport of nutrients across the choroid plexus. Micro Res Tech.

[5] Gardner D, Lucas P, Cowdry R (1990). CSF metabolites in borderline personality disorder compared with normal controls. Biologic Psych.

[6] Iliff J, Wang M, Liao Y, Plogg B, Peng W, Gundersen G (2012). A paravascular pathway facilitates CSF flow through the brain parenchyma and the clearance of interstitial solutes, including amyloid β. Sci Trans Med..

[7] Xie L, Kang H, Xu Q, Chen M, Liao Y, Thiyagarajan M (2013). Sleep drives metabolite clearance from the adult brain. Science.

[8] Lundberg N. The saga of the Monro-Kellie doctrine. In: Ishii S, Nagel H, Brock M, eds. Intracranial Pressure V. Berlin: Springer; 1983. p. 68–76.

[9] Baledent O, Gondry-Jouet C, Meyer ME, De Marco G, Le Gars D, Henry- Feugeas MC (2004). Relationship between cerebrospinal fluid and blood dynamics in healthy volunteers and patients with communicating hydrocephalus. Invest Radiol.

[10] Fultz N, Bonmassar G, Setsompop K, Stickgold R, Rosen B, Pollmeni J (2019). Coupled electrophysiological, hemodynamic, and cerebrospinal fluid oscillations in human sleep. Science.

[11] Dreha-Kulaczewski S, Joseph A, Merboldt K-D, Ludwig H-C, Gartner J, Frahm J (2015). Inspiration is the major regulator of human CSF flow. J Neurosci.

[12] Vinje V, Ringstad G, Lindstrom E, Valnes L, Rognes M, Eide P, Mardal K-A (2019). Respiratory influence on cerebrospinal fluid flow—a computational study based on long-term intracranial pressure measurements. Sci Rep.

[13] Carare R, Aldea R, Bulters D, Alzetani A, Birch A, Richardson G (2020). Vasomotion drives periarterial drainage of Aβ from the brain. Neuron.

[14] Holmlund P, Johansson E, Qvarlander S, Wahlin A, Ambarki K, Koskinen LOD (2017). Human jugular vein collapse in the upright posture: implications for postural intracranial pressure regulation. Fluids Barriers CNS.

[15] Gehlen M, Kurtcuoglu V, Schmid Daners M (2017). Is posture-related craniospinal compliance shift caused by jugular vein collapse? A theoretical analysis. Fluids Barriers CNS.

[16] Stephensen H, Tisell M, Wikkelso C (2002). There is no transmantle pressure gradient in communicating or noncommunicating hydrocephalus. Neurosurg.

[17] Eide PK, Saehle T (2010). Is ventriculomegaly in idiopathic normal pressure hydrocephalus associated with a transmantle gradient in pulsatile intracranial pressure?. Acta Neurochir.

[18] Ringstad G, Lindstrom EK, Vatnehol S, Mardal K-A, Emblem KE, Eide PK (2017). Non-invasive assessment of pulsatile intracranial pressure with phase-contrast magnetic resonance imaging. PLoS ONE.

[19] Buell T, Heiss J, Oldfield E (2015). Pathogenesis and cerebrospinal fluid hydrodynamics of the Chiari I malformation. Neurosurg Clin North Amer.

[20] Pickard J, Coleman M, Czosnyka M (2005). Hydrocephalus, ventriculomegaly and the vegetative state; a review. Neuropsych Rehab.

[21] Milhorat T, Capocelli A, Kotzen R, Bolognese P, Heger I, Cottrell J (1997). Intramedullary pressure in syringomyelia: clinical and pathophysiological correlates of syrinx distension. Neurosurgery.

[22] Jones H (1978). The roof of the fourth ventricle in amphibian brains. J Zool London.

[23] Milsom W (1991). Intermittent breathing in vertebrates. Ann Rev Physiol.

[24] Douse M, Mitchell G (1992). (1992) Episodic breathing in alligators: role of sensory feedback. Resp Physiol.

[25] Farmer C, Carrier D (2000). Pelvic aspiration in the American alligator (*Alligator mississippiensis*). J Exp Biol.

[26] Klassen M, Adams J, Cramberg M, Knoche L, Young BA (2020). The narial musculature of *Alligator mississippiensis*: Can a muscle be its own antagonist?. J Morphol.

[27] Axelsson M, Franklin CE, Lofman CO, Nilsson S, Grigg GC (1996). Dynamic anatomical study of cardiac shunting in crocodiles using high-resolution angioscopy. J Exp Biol.

[28] Syme DA, Gamperl K, Jones D (2002). Delayed depolarization of the cog-wheel valve and pulmonary-to-systemic shunting in alligators. J Exp Biol.

[29] Knoche L, Young BA, Kondrashova T (2019). The influence of gravitational gradients on the American alligator (*Alligator mississippiensis*). Anat Physiol Curr Res..

[30] Kondrashova T, Blanchard J, Knoche L, Potter J, Young BA (2019). Intracranial pressure in the American alligator (*Alligator mississippiensis*): reptilian meninges and orthostatic gradients. J Comp Physiol A..

[31] Kasprowicz M, Lalou D, Czosnyka M, Garnett M, Czosnyka Z (2016). Intracranial pressure, its components and cerebrospinal fluid pressure-volume compensation. Acta Neurol Scand.

[32] Klarica M, Kuzman T, Mandac I, Rados M, Oreskovic D, Bulat M (2007). The effect of body position on intracranial and intraocular pressure in cats. Period Biolog.

[33] Lillywhite H (1996). Gravity, blood circulation, and the adaptation of form and function in lower vertebrates. J Exp Zool A.

[34] Young BA, Wassersug R, Pinder A (1997). Gravitational gradients and blood flow patterns in specialized arboreal (*Ahaetulla nasuta*) and terrestrial (*Crotalus adamanteus*) snakes. J Exp Zool.

[35] Marmarou A, Shulman K, LaMorgese J (1975). Compartmental analysis of compliance and outflow resistance of the cerebrospinal fluid system. J Neurosurg.

[36] Czosnyka M, Pickard J (2004). Monitoring and interpretation of intracranial pressure. J Neurol Neurosurg Psych.

[37] Satchell G, Rossiter G (1972). Pulsatile pressures in the cranial fluids of *Heterodontus portusjacksoni*. J Exp Biol.

[38] Guinane J (1975). Cerebrospinal fluid pulse pressure and brain compliance in adult cats. Neurology.

[39] Malkinson T, Veale W, Cooper K (1978). Measurement of intracranial pressure in the unanesthetized rabbit. Brain Res Bull.

[40] Guthrie T, Dunbar H, Karpell B (1970). Ventricular size and chronic increased intracranial venous pressure in the dog. J Neurosurg.

[41] Wood J, Poplack D, Flor W, Gunby N, Ommaya A (1977). Chronic ventricular cerebrospinal fluid sampling, drug injections, and pressure monitoring using subcutaneous reservoirs in monkeys. Neurosurgery.

[42] Moore R, Trim C (1993). Effect of hypercapnia or Xylazine on lateral ventricle and lumbosacral cerebrospinal fluid pressures in pentobarbital-anesthetized horses. Vet Surg.

[43] Oshio K, Watanabe H, Song Y, Verkman A, Manley G (2004). Reduced cerebrospinal fluid production and intracranial pressure in mice lacking choroid plexus water channel Aquaporin-1. FASEB J..

[44] Dunbar H, Guthrie T, Karpell B (1966). A study of cerebrospinal fluid pulse wave. Arch Neurol.

[45] Wagshul M, Eide P, Madsen J. The pulsating brain: a review of experimental and clinical studies of intracranial pulsatility. Fluids Barriers CNS 2011; http://www.fluidsbarrierscns.com/content/8/1/5 10.1186/2045-8118-8-5 PMC3042979 21349153

[46] Burda D (1969). Developmental aspects of intracranial arterial supply in the alligator brain. J Comp Neurol.

[47] Porter W, Sedlmayr J, Witmer L (2016). Vascular patterns in the heads of crocodilians: blood vessels and sites of thermal exchange. J Anat.

[48] Franklin C, Axelsson M (1994). The intrinsic properties of an in situ perfused crocodile heart. J Exp Biol.

[49] Franklin C, Axelsson M (2000). Physiology: an actively controlled heart valve. Nature.

[50] Young B, Adams J, Segal S, Kondrashova T (2018). Hemodynamics of tonic immobility in the American alligator (*Alligator mississippiensis*) identified through Doppler ultrasonography. J Comp Physiol A.

[51] Antoni N (1946). Pressure curves from the cerebrospinal fluid. Acta Med Scand.

[52] Greitz D, Wirestam R, Franck A, Nordell B, Thomsen C, Stahlberg F (1992). Pulsatile brain movement and associated hydrodynamics studied by magnetic resonance phase imaging. Neuroradiology.

[53] Bering E (1955). Choroid plexus and arterial pulsations of cerebrospinal fluid. Arch Neurol Psychiat.

[54] Williams B (1976). Cerebrospinal fluid pressure changes in response to coughing. Brain.

[55] Bhadelia R, Patz S, Hellman C, Khatami D, Kasoer E, Zhao Y (2016). Cough-associated changes in CSF flow in Chiari I malformation evaluated by real-time MRI. Amer J Neuroadiol.

[56] Guerci A, Shi A, Levin H, Tsitlik J, Weisfeldt M, Chandra N (1985). Transmission of intrathoracic pressure to the intracranial space during cardiopulmonary resuscitation in dogs. Circ Res.

[57] Marino B, Yannopoulos D, Sigurdsson G, Lai L, Cho C, Redington A (2004). Spontaneous breathing through an inspiratory impedence threshold device augments cardiac index and stroke volume index in a pediatric porcine model of hemorrhagic hypovolemia. Crit Care Med.

[58] Agostoni E (1961). A graphical analysis of thoracoabdominal mechanics during the breathing cycle. J Appl Physiol.

[59] Claessens L (2009). A cineradiographic study of lung ventilation in *Alligator mississippiensis*. J Exp Zool A.

[60] Young B, Adams J, Beary JM, Mardal K-A, Schneider R, Kondrashova T (2020). The myodural bridge of the American alligator (*Alligator mississippiensis*) alters CSF flow. J Exp Biol.

[61] Brocklehurst R, Moritz S, Codd J, Sellers W, Brainerd E (2017). Rib kinematics during lung ventilation in the American alligator (*Alligator mississippiensis*): an XROMM analysis. J Exp Biol.

[62] Gatson B, Goe A, Granone T, Wellehan J (2017). Intramuscular epinephrine results in reduced anesthetic recoverytime in American alligators (*Alligator mississippiensis*) undergoing isoflurane anesthesia. J Zoo Wild Med.

[63] Taylor E, Leite C, Skovgaard N (2010). Autonomic control of cardiorespiratory interactions in fish, amphibians, and reptiles. Brazilian J Med Biol Res.

[64] Bloomfield G, Ridlings P, Blocher C, Marmarou A, Sugerman H (1997). A proposed relationship between increased intra-abdominal, intrathoracic, and intracranial pressure. Crit Care Med.

[65] Geregele L, Baledent O, Manet R, Lalou A, Barszcz S, Kasprowicz M, Miller K (2019). Dynamics of cerebrospinal fluid: From theoretical models to clinical applications. Biomechanics of the Brain.

[66] Young B, Potter J, Blanchard J, Knoche L, Kondrashova T (2020). Cardiac response to stimulation and stress in the American alligator (*Alligator mississippiensis*). Amph-Rept.

[67] Magnaes B (1989). Clinical studies of cranial and spinal compliance and the craniospinal flow of cerebrospinal fluid. British J Neurosurg.

[68] Hentschel S, Mardal K-A, Lovgren AE, Linge S, Haughton V (2010). Characterization of cyclic CSF flow in the foramen magnum and upper cervical spinal canal with MRI flow imaging and computational fluid dynamics. Amer J Neuroradiol.

[69] Dreha-Kulaczewski S, Joseph A, Merboldt K-D, Ludwig H-C, Gartner J, Frahm J (2017). Identification of the upward movement of human CSF *in Vivo* and its relation to the brain venous system. J Neurosci.

[70] Olstad E, Ringers C, Hansen J, Wens A, Brandt C (2019). Ciliary beating compartmentalizes cerebrospinal fluid flow in the brain and regulates ventricular development. Curr Biol.

[71] Thouvenin O, Keiser L, Cantaut-Belarif Y, Carbo-Tano M, Verweij F, Jurisch-Yaksi N (2020). Origin and role of the cerebrospinal fluid bidirectional flow in the central canal. eLIFE.

[72] Eide PK, Sorteberg A, Sorteberg W, Lindstrom E, Mardal K-A, Ringstad G (2019). "Bucket" cerebrospinal fluid bulk flow: when the terrain disagrees with the map. Acta Neurochir.

[73] Kasuga Y, Nagai H, Hasegawa Y, Nitta M (1987). Transmission characteristics of pulse waves in the intracranial cavity of dogs. J Neurosurg.

[74] Piper I, Miller J, Dearden N, Leggate J, Robertson I (1990). Systems analysis of cerebrovascular pressure transmission: an observational study in head-injured patients. J Neurosur.

[75] Stark D, Gans C, Northcutt R, Ulinski P (1979). Cranio-cerebral relations in recent reptiles. Biology of the reptilia.

[76] Zippel K, Lillywhite H, Mladinich C (2003). Anatomy of the crocodilian spinal vein. J Morphol.

[77] Alperin N, Hushek S, Lee S, Sivaramakrishnan A, Lichtor T (2005). MRI study of cerebral blood flow and CSF flow dynamics in an upright posture: the effect of posture on the intracranial compliance and pressure. Acta Neurochir.

[78] Alperin N, Mazda M, Lichtor T, Lee S (2006). From cerebrospinal fluid pulsations to noninvasive intracranial compliance and pressure measured by MRI flow studies. Curr Med Imag Rev.

[79] Williams B (1981). Simultaneous cerebral and spinal fluid pressure recordings. Acta Neurochir.

[80] Behrens A, Lenfeldt N, Qvarlander S, Koskinen L-O, Malm J, Eklund A (2013). Are intracranial pressure wave amplitudes measurable through lumbar puncture?. Acta Neurol Scand.

[81] Vinje V. Simulating cerebrospinal fluid flow and spinal cord movement associated with syringomyelia. Ms Thesis, Department of Mathematics, University of Oslo. 2016.

